# Distress or no distress, that's the question: A cutoff point for distress in a working population

**DOI:** 10.1186/1745-6673-3-3

**Published:** 2008-01-18

**Authors:** Willem van Rhenen, Frank JH van Dijk, Wilmar B Schaufeli, Roland WB Blonk

**Affiliations:** 1 Academic Medical Center, Coronel Institute of Occupational Health, University of Amsterdam, Amsterdam, The Netherlands; 2 Department of Occupational Health Services, ArboNed Utrecht, Utrecht, The Netherlands; 3 Utrecht University, Department of Psychology and Research Institute Psychology & Health, Utrecht, The Netherlands; 4 TNO Work and Employment, Hoofddorp, The Netherlands

## Abstract

**Background:**

The objective of the present study is to establish an optimal cutoff point for distress measured with the corresponding scale of the 4DSQ, using the prediction of sickness absence as a criterion. The cutoff point should result in a measure that can be used as a credible selection instrument for sickness absence in occupational health practice and in future studies on distress and mental disorders.

**Methods:**

Distress is measured using the Four Dimensional Symptom Questionnaire (4DSQ), a 50-item self-report questionnaire, in a working population with and without sickness absence due to distress. Sensitivity and specificity were compared for various potential cutoff points, and a receiver operating characteristics analysis was conducted.

**Results and conclusion:**

A distress cutoff point of ≥11 was defined. The choice was based on a challenging specificity and negative predictive value and indicates a distress level at which an employee is presumably at risk for subsequent sick leave on psychological grounds. The defined distress cutoff point is appropriate for use in occupational health practice and in studies of distress in working populations.

## Background

Distress is a heterogeneously defined and imprecise term that refers to unpleasant subjective stress responses [1]. Verhaak [2] estimated the prevalence in the general population in western communities as 15–25%. In a clinical population of cancer patients, Keller et al. [3] reported clinically relevant distress in about 25% of patients (across other studies this figure ranges from 5% to 50%). In the working population, Bültmann et al. [4] documented a prevalence of psychological distress as 21.8% for men and 25.9% for women Distress and stress-related disorders are widespread among working and non-working populations and are responsible for high costs in terms of human suffering, disability and economic losses.

Despite the high prevalence and costly consequences, distress still goes unrecognized by health professionals. In clinical settings comparing the patient-reported distress to the doctor's rating, the vast majority of the cases go unrecognized [5]. Although figures for occupational health physicians are unknown, we assume that these will be similar to those in clinical settings.

The underrating of distress is not surprising since in health care the focus is not on distress, but on depression and anxiety disorders and their consequences. Contrary to distress, both disorders seem well-defined [6-9]. Both are highly prevalent, contributing to almost 13% of the total world disease burden [10,11], ranging in different studies from 12% to 49% for one-year prevalence rates and lifetime prevalence for depression, and from 8% to 29% [12-15] for anxiety disorders. Measured using the Hospital Anxiety and depression (HAD) Scale in the Netherlands, the one-year prevalence of depression and anxiety in the working population is 7.1% and 8.2% for males and 6.2% and 10% for females [16].

For the recognition, prevention and treatment of mental health problems, the underestimation of distress can be regarded as unfavorable for several reasons.

The first reason is the imminent concomitance of distress and sickness absence. Distress as a main cause of sickness absence can be labeled under 'adjustment disorders' following the DSM IV classification [17]. In the Netherlands, approximately 30% of the employees who visit the occupational physician for sickness absence report mental health problems [18] including common mental health problems like adjustment disorders, but also psychiatric disorders such as anxiety and depressive disorders. The majority of the employees absent for mental health reasons can be classified as having an adjustment disorder [19]. Nieuwenhuijsen et al. [20] demonstrated a percentage of 59% in employees absent for mental health problems. Prevention of – at least a part – of sickness absence through a reduction in high levels of distress is a challenge for the occupational health professional and can be a benefit for employees and companies.

A second reason for a focus on distress is the high concurrence with anxiety and mood disorders [21-23], which in turn show a high degree of intercorrelation [24-26]. Distress symptoms such as concentration problems, irritability and fatigue are common to both anxiety and depression in the DSM IV diagnostic criteria [27].

A third reason for discerning distress is the implication for treatment and guidance. The reduction of distress presumably has its own typical approach. In the past, 20% of patients reporting themselves sick with an adjustment disorder due to distress did not return to work within one year [28]. Van der Klink et al [17] demonstrated that an activating intervention based on the principles of time contingency and cognitive behavioral treatment was successful in reducing sick leave duration by 25–30% compared with 'care as usual'. Another study [29] among working employees showed that specific (preventive) cognitive and physical interventions are equally effective in reducing distress levels by 50–60%.

In the last two decades, several questionnaires have been developed to measure distress. The Mood and Anxiety Symptom Questionnaire (MASQ) established by Watson and Clark [9] and the Depression Anxiety Stress Scale (DASS) originated by Lovibond and Lovibond [30] are based on the tripartite model of Clark and Watson [9]. Recently, Terluin [7] introduced the Four Dimensional Questionnaire (4DSQ) developed to differentiate distress from two psychiatric illnesses (depression and anxiety) and from somatization. Together, these four symptom clusters account for the majority of the mental health problems in primary health care. According to Terluin, distress is the psychological squeal of strain caused by unsuccessfully coping with a stressor. Stressors can be the common cause for distress and depression or anxiety. Under less favorable conditions, distress might be a precursor for more serious psychiatric disorders. On the other hand, psychiatric illness can act as a stressor that aggravates strain and distress. That may explain why individuals with depression and anxiety in many cases also exhibit distress.

The 4DSQ, a 50-item self-report questionnaire, has been developed for clinical and non-clinical populations with psychological complaints and has been validated in primary health care [31,32] and in occupational health care [7,29]. The four scales of the DSQ are internally consistent, with Cronbach's alphas ranging from .79 to .90. The subscale distress, the focus of this study, is associated with job stressors and indicators of strain, which supports the utility of the questionnaire for screening purposes. Since working employees with a high rate of distress as a consequence of job stressors and strain, run a high risk of sickness absence, a cutoff point for distress can be helpful for the identification – and maybe even monitoring – of employees at risk for sickness absence and for the selection of cases for support like stress management programs or treatment in order to prevent absenteeism.

The use of a cutoff point [4,33] for inclusion in preventive stress management programs has remarkably not been reported until now. Because of the size of the problem, reducing sickness absenteeism by applying interventions to reduce work-related stress is of great importance. Individually focused programs aim to increase the employee's mental resilience [34], usually referred to as a stress management training [35,36]. And although the term stress management training may suggest a rather uniform set of intervention strategies, it usually refers to a mixture of treatment techniques. To a certain extent these (work-related) stress interventions claim to reduce psychological complaints [37-40], to increase individual quality of life [41-43], to reduce stress-related health care costs [44,45]. and to reduce absenteeism [46-48]. Although such effects of stress management interventions have been shown, the effects on absenteeism are still subject to debate. Differences between the intervention programs as well as methodological differences between these studies – such as the lack of a control group, inadequate collection of data or different study designs with different measures – are brought forward to explain these inconsistent results. However, another important cause may be the lack of a cutoff point in most studies for selecting participants [34]. It is a lamentable omission for current stress management programs and guidelines that we miss clear criteria for the referral of employees with a certain level of distress to occupational health physicians or psychosocial care teams.

In addition, the distress dimension of the 4DSQ and a cutoff point can be used as a valid estimator for the prevalence of distress across demographic and occupational subgroups [29]. A well-founded cutoff point can be used as a criterion to classify cases for research purposes. "Cutoff scores are used in a wide variety of settings to divide a score scale or other set of data into two or more categories, with inferences made or actions taken on the basis of this classification" as has been stated by Dwyer [49]. The choice of such a categorization represented by one or more cutoff points, however, is a result of judgments. One of the unwanted side-effects of this process of decision making may be the emergence of different cutoff points in different studies [50]. This makes comparisons across studies extremely difficult or even impossible.

Consequently, clarification of the process of decision making is indispensable. In this article we therefore describe explicitly the process by which we selected an optimal cutoff score of a risk factor that gives the best separation between employees with high distress levels related to the risk for subsequent sickness absence due to psychological complaints on the one hand, and employees who are not at risk on the other. By doing this, the results of this study can be compared with the results of other studies.

In conclusion, the objective of the present study is to establish an optimal cutoff point for distress measured using the corresponding scale of the 4DSQ, with the prediction of sickness absence as a criterion. The cutoff point should result in a measure that can be used as a credible selection instrument for stress management programs or other interventions to prevent sickness absence due to psychological complaints in occupational health practice and in future studies on distress and mental disorders.

## Method

### Sample

Two samples of employees with presupposed differences in distress were used. Both employee samples worked in a large telecom company in the Netherlands and were approached by the company's Department of Occupational Health.

The first sample, representing the 'healthy working employees', were participants in an occupational health survey with a focus on occupational stress. Questionnaires were mailed to all employees of the company (N = 7,522). The questionnaires were completed by 3,852 employees (response rate 51%). The sample consisted mainly of men (91%), medium- or highly-educated employees (74%), and had a mean age of 43.9 years. At the moment at which the employees filled in the questionnaire, 247 (6.4%) were on sick leave; these were excluded from the sample resulting in 3605 employees.

The second sample consisted of 280 employees who had been on sick leave for at least two weeks and, in accordance with the procedure, were referred to their occupational physician. To be included in the sample, employees had to be on their first sickness leave because of stress at work or a stress-related disorder due to a recent identifiable psychosocial stressor at work. The employees had to demonstrate at least eight out of 16 distress symptoms of the 4DSQ scale (at level one or higher) that represent the main symptom categories of the DSM IV adjustment disorder [17]. Exclusion criteria were a psychiatric diagnosis such as an anxiety disorder or a depressive disorder and physical co-morbidity.

### Measure

The 4DSQ is a 50-item self-report questionnaire [7] that identifies four symptom dimensions: distress (16 items, e.g. "Did you feel easily irritated?"), depression (6 items, e.g. "Did you feel that you can't enjoy anymore?"), anxiety (12 items, e.g. "Were you afraid of anything when there was really no need for you to be afraid?") and somatization (16 items, e.g. "Did you suffer from excessive perspiration?"). Participants are instructed to indicate how they felt during the previous week, and the items are scored on a 5-point Likert scale (from 0 = 'No' to 4 = 'Very often'). In the application of the 4DSQ, to reduce the influence of aggravating response tendencies on sum scores, all item scores of '3' and '4' are recoded into a score of '2' before calculating sum-scores per dimension. Thus, symptoms are rated as absent ('no': 0 points), doubtfully present ('sometimes': 1 point) or present at a clinically significant level ('regularly/often/very often': 2 points). The factor score for distress ranges from 0–32, where a high scores indicates substantial distress. The value for Cronbach's alpha for distress is .90.

### Analyses

Distress scores (means, standard deviations and percentile scores) were calculated for both samples. Considering the aim of identifying employees in a working environment at risk for sickness absence due to psychological complaints, and applying the recommendations of Dwyer [48], we first explore the test threshold which can discriminate well between distressed employees without sickness absence due to psychological problems and employees on sick leave because of stress or a stress-related disorder. A Receiver Operating Characteristic (ROC) analysis was used to define a cutoff point, displaying the predicted probability of the target event – sickness absence. The ROC shows a range of cutoff points with corresponding sensitivity and specificity.

To find with the ROC analysis the most optimal cutoff point that discriminates best between both groups, we formed in the first place a purposefully created artificial study population with an equal number of employees from both samples: 280 'healthy working employees' randomly selected from the first sample, and in addition the total second sample of 280 employees on sick leave. Together, both populations form what we called the 'equal sample study population', in total 560 employees.

Secondly and in order to check, using a ROC analysis, the described ROC curve and its cutoff point in a representative population, we formed a second artificial study population similar to a working population with a normal prevalence of sickness absence due to psychological complaints (2%). Therefore we added 72 employees (2% of 3605) randomly selected from the population on sick leave for psychological reasons, to the 3605 healthy working employees, thus adhering better to the conditions in practice. This study population is called the 'representative study population'.

For use in occupational health practice, it is not only important to find nearly all employees-at-risk, but also to exclude false-positive employees. Therefore, during the evaluation, the establishment of an optimal cutoff point is based on an optimal trade-off between sensitivity and specificity. In a screening situation, however, where the prevalence of absence due to distress is low (2%), specificity is more crucial than sensitivity. Increasing the specificity at the expense of sensitivity will lead to a substantial increase of the positive predictive value, while the considerable reduction in sensitivity decreases the negative predictive value only marginally. Beforehand, we had made the choice to set specificity at above 90%. Applied to a working population, a screening test with this specificity can exclude a large majority of persons *not *at risk.

## Results

The demographics are specified in Table 1. For the first sample, questionnaires were mailed to all employees of the company (N = 7,522). The questionnaires were completed by 3,852 employees (response rate 51%). The sample consisted mainly of men (91%), medium-or highly-educated employees (74%), and had a mean age of 43.9 years. At the moment at which the employees filled in the questionnaire 247 (6.4%) employees were on sick leave; these were excluded from the sample. The second sample (n = 280) who had been on sick leave for at least two weeks because of stress or stress-related disorder, consisted of 66% men, 66% medium- or highly-educated employees, and the mean age was 41.9 years.

**Table 1:** Characteristics of the samples 'healthy working employees' and 'employees on sick leave due to psychological complaints'

	Age			Gender	Marital status	Level of education		
Sample	n	Mean years	SD	% Female	% Married	% Low	% Medium	% High
Healthy working employees	3605	43.9	8.1	9	78	26	49	25
Employees on sick leave due to psychological complaints	280	41.9	8.1	34	66	34	41	25

Table 2 shows the means, standard deviations, and the percentile scores of distress for both samples (N = 3605 and N = 280). As expected, employees on sickness absence due to psychological complaints scored significantly higher on distress (Mean = 22.3, SD = 6.7) than the sample of healthy working employees (Mean = 4.0, SD = 5.0) (T-test; p <.000).

**Table 2:** Levels of distress (mean and SD score on 4DSQ distress scale) in the samples of 'healthy working employees' and 'employees on sick leave due to psychological complaints'

		Distress (range 0–32)						
				Percentile				
Sample	*n*	Mean	SD	5	25	50	75	95
Healthy working employees	3605	4.0	5.0	0.0	0.0	2.0	6.0	14.0
Employees on sickness absence due to psychological complaints	280	22.3	6.7	9.0	18.0	24.0	28.0	31.0

As can be seen from Table 3, the optimal cutoff score for distress, given a specificity that exceeds 90%, equals 10 in the equal sample study population (N = 560). As expected, in the case of the representative study population (N = 3677), the cutoff score equals 11, with the same restriction for specificity. Table 2 shows that the cutoff score of 11 is located between the 75^th ^and 95^th ^percentile of the distribution of distress scores in the population of healthy working employees: most healthy employees have less distress. In the sample of employees on sickness absence, this cutoff score is located close to the 5^th ^percentile, which means that the overall majority exceeds this distress level in this population.

**Table 3:** Sensitivity and specificity of alternative cutoff points in the 'equal sample population' and the 'representative sample population'

Equal sample population (n = 560): 280 healthy working employees plus 280 employees on sick leave for psychological complaints			Representative sample population (n = 3677): 3605 healthy working employees plus 72 employees on sick leave for psychological complaints		
Cutoff	Sensitivity	Specificity	Cutoff	Sensitivity	Specificity
7.50	.979	.854	7.50	.973	.830
8.50	.975	.882	8.50	.973	.863
**9.50**	**.950**	**.893**	9.50	.945	.884
10.50	.939	.914	**10.50**	**.945**	**.905**
11.50	.929	.921	11.50	.932	.918
12.50	.907	.936	12.50	.890	.932
13.50	.882	.954	13.50	.877	.945

In the representative study population (N = 3677), a cutoff point equal to or higher than 11 has as a consequence that 69 of 72 absent employees are correctly classified as being absent due to psychological complaints, corresponding with a sensitivity of 95%. Within the population of 3605 employees without sickness absence, 3261 employees are classified as not distressed, corresponding with a specificity of 90%. The positive predictive value is .17, whereas the negative predictive value is .998. In addition, Table 3 shows the sensitivity and specificity of alternative cutoff points.

**Figure 1:**
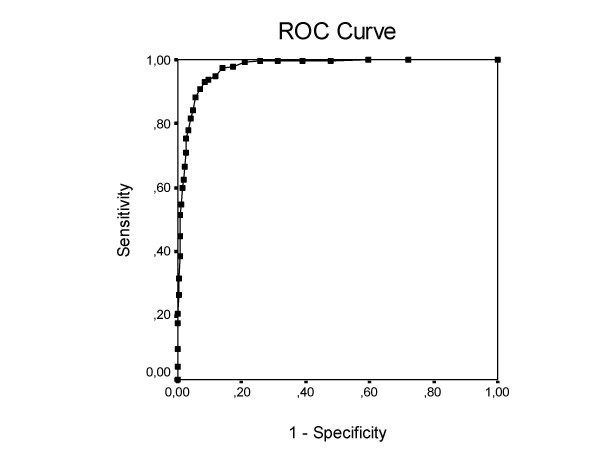
The receiver operating characteristic curve of Distress total scores representing potential cutoff points in the representative sample. Area under the curve = .975.

The Area Under the ROC Curve (AUC) statistic (Fig 1) has been obtained by comparing the full range of possible cutoff scores. The area under the curve was 0.98, which is excellent, because in that case the positive likelihood ratio (LR+: the probability to find a positive test result in stressed employees compared with employees who are not stressed) is 10 or more and the negative likelihood ratio (LR-: the probability to find a negative test result in stressed employees compared with employees who are not stressed) is 0.1 or less. This means that employees who score above the chosen cutoff score are far more likely to report sick compared with employees who score under the cutoff score.

## Discussion

In the present study, a cutoff point ≥ 11 was chosen for the distress scale of the 4DSQ to measure distress in a working population. This cutoff point corresponds with a sensitivity of 95% and a challenging specificity of 90% and negative predictive value of .998, and indicates a distress level that puts an employee "at risk" for subsequent sick leave on psychological grounds.

Two issues require some discussion here. One issue is that we used as our study population employees working for a telecom company, which in potential restricts the generalizability of the cutoff point to other working samples. Therefore, we recommend that more studies be undertaken with a clear reference to the populations studied.

The second issue that should be kept in mind when implementing the results of this paper is that psychological complaints range from zero to many, therefore distress can be best viewed as a continuum as opposed to a dichotomy. Applying a cutoff point to this continuum potentially reduces information [51]. If the purpose of a study is to explore the etiology of distress, it is more informative to use a range of distress scores. A dichotomy, however, is useful when the prevalence of distress has to be compared in different subgroups or when employees have to be selected for stress management or treatment.

Unfortunately, there is no other study to compare with, which reported a cutoff point based on the AUC statistic for identifying cases of sickness absence related to distress in a working population. It is noteworthy that the use of a cutoff point for inclusion in preventive stress management programs has not often been reported until now. Moreover, in the meta-analysis of van der Klink et al [34], only four studies out of forty-eight involved participant selection with regard to high baseline stress levels.

The choice of a cutoff point of 11 results in a measure that can be used as a cutoff point in future studies on distress and mental disorders, and is appropriate for use in occupational health practice as a credible selection instrument for stress management or other interventions to prevent sickness absence.

The cutoff point of 11 corresponds with a sensitivity of 95% and a specificity of 90% in a representative study population as created (a population with 2% sickness absence due to psychological complaints). The positive predictive value of the cutoff point in this study population is 17%, whereas the negative predictive value is 99.8%. This means that there is a one in six chance that an employee in a working population who scores on or above the cutoff score of 11 may really turn out to go on sick leave for psychological reasons. On the other hand, in case of a negative test outcome there is only a two-tenths of a percent chance of a false negative result. This issue is mentioned by Dwyer [49] as the problem of 'misclassification' as an inevitable consequence of dividing a sample of employees into those at risk and not at risk.

The occupational health physician can be confident that the employee is actually free of the chance to be absent for psychological problems when the test result is negative. On the other hand, the large majority of the selected population with a cutoff point greater than 11 does not belong to the population on sick leave, which is a reason for further considerations. In our opinion this finding may be acceptable. Since the 4DSQ is inexpensive, easy to administer, poses little risk and causes minimal discomfort for the employee, the overestimation of positive results can be corrected by embedding the test procedure in a broader program that includes a further study of each positive finding. A second test, for example an individual interview, can distinguish more precisely whether an employee needs an intervention or not. This serial multi testing [52] is quite popular in the regular health care field, and can also be implemented in the practice of occupational health care.

Furthermore, an argument in favor of the application of the chosen cutoff point is the assumption that the induction of interventions can useful for all stressed employees. Interventions based on a physically-oriented approach like relaxation and physical exercise aim at improving mental health by reducing physiological arousal [38]. There is good evidence from randomized controlled trials that relaxation techniques can reduce psychological complaints related to stressful situations [53]. Positive effects of cognitively-oriented interventions have been reported extensively [34]. Changing appraisal processes and enhancing coping skills are the fundaments for coping with stress more effectively. Therefore, learning a method for managing demands and stressors, and altering how one responds to inevitable and necessary demands will benefit employees instead of harming them. One issue to discuss is the effect of labeling on public attitudes toward people with stress. Angermeyer and Matschinger [54] found out that labeling people with mental problems has an impact on public attitudes only if there is particularly a link with the stereotype of dangerousness (e.g. schizophrenia). By contrast, 'distress', denoting a wide range of mental health problems, is generally accepted by the public and therefore not perceived as a danger. A critical note might be that, in some companies, labeling can be a problem, especially during periods of downsizing [55,56].

Finally, there is an issue of costs. In our opinion, a program for screening a working population using the 4DSQ, including interventions, is far less expensive than sickness absence due to psychological problems. Costs due to the consequences of stress in the Netherlands are estimated at 6.1 billion Euros a year (TNO), 2.7 billion of which is due to sickness absence and allowances. This is comparable with over 1% of the Gross National Product of the Netherlands.

## Conclusion

A distress cutoff of ≥ 11 was defined. This cutoff point will result in a measure that can be used as a credible selection instrument for interventions such as stress management programs to reduce distress and sickness absence due to psychological complaints in occupational health practice and as a well-founded cutoff point in future studies on distress and mental disorders.

## Competing interests

The author(s) declare that they have no competing interests.

## Authors' contributions

WvR conceived and designed in consultation with the other authors the study, collected and analyzed the data and drafted the manuscript; FJHvD contributed to the concept and design and drafted the manuscript; WBS contributed to the concept and design and drafted the manuscript; RWBB contributed to the concept and design, analysis of the data and drafted the manuscript. All authors read and approved the final manuscript.
